# Drug target prediction from perturbation transcriptomics via a biological function-guided hypergraph siamese network

**DOI:** 10.1093/bioinformatics/btag475

**Published:** 2026-08-06

**Authors:** Xinyi Zhang, Xinliang Sun, Jiuxu Yang, Min Li

**Affiliations:** School of Computer Science and Engineering, Central South University, Changsha 410083, China; School of Computer Science and Engineering, Central South University, Changsha 410083, China; School of Computer Science and Engineering, Central South University, Changsha 410083, China; School of Computer Science and Engineering, Central South University, Changsha 410083, China

## Abstract

**Motivation:**

Understanding how small molecules modulate cellular states remains a critical challenge in drug discovery. The advent of perturbation transcriptomics offers new avenues for elucidating drug-target interactions by capturing cellular transcriptional responses to perturbations.

**Results:**

In this study, we propose BioHSNet, a biological function-guided hypergraph siamese network for inferring drug-target interactions from perturbation transcriptomics. BioHSNet utilizes hyperedge representations of functionally grouped gene expression to capture higher-order functional relationships, and integrates compound structural information into the model to bridge chemical structure and functional response. Experimental results demonstrate that BioHSNet outperforms other transcriptome-based methods on the Broad Institute’s L1000 datasets, particularly in cold start scenarios. The case study further demonstrates its practical utility for target prediction and drug screening.

**Availability and Implementation:**

The source code is available at https://github.com/Zxinyizhang/BioHSNet.

## 1 Introduction

Accurately predicting drug-target interactions (DTIs) is a pivotal step in drug discovery, with significant implications for understanding drug mechanisms, facilitating drug repurposing, and accelerating the development of novel therapeutics (Bai *et al.* 2023, Lu *et al.* 2025). However, traditional computational pipelines, including ligand-based and structure-based approaches, have intrinsic limitations (Yang *et al.* 2021). The assumption that structural similarity implies similar bioactivity is often violated by small chemical modifications (Qiao *et al.* 2025). In addition, structure-based modeling depends on high-resolution protein structures and computationally intensive simulations, which constrain scalability and target coverage. These constraints have catalyzed a shift toward phenotype-based strategies that prioritize cellular responses over predefined targets, aiming to capture drug action in a more holistic and mechanism-agnostic manner (Pham *et al.* 2021, Tong *et al.* 2024, Akwaronwu *et al.* 2026).

The advent of perturbation transcriptomics offers new avenues for elucidating DTIs by capturing cellular transcriptional responses to perturbations (Tanoli *et al.* 2025, Zhao *et al.* 2026). For example, the Library of Integrated Network-Based Cellular Signatures (LINCS) L1000 dataset (Subramanian *et al.* 2017) has generated expression signatures for over 20 000 small molecules and gene perturbations across diverse cell lines. Connectivity Map (CMap) (Subramanian *et al.* 2017) infers the likely targets of a new compound by comparing the similarity of its gene-expression signature to reference signatures. ProTINA (Noh *et al.* 2018) integrates gene expression profiles with gene regulatory networks and uses a dynamic modeling framework to infer drug targets from differential expression data. However, both methods suffer from limited computational efficiency and relatively poor predictive accuracy. The method by Pabon *et al.* (2018) explores associations between compound and gene perturbations and uses Random Forest to predict drug targets, but it still falls short in capturing complex dependencies. In contrast, PertKGE (Ni *et al.* 2024) and KGDRP (Ye *et al.* 2025) enhance prediction accuracy by leveraging knowledge graphs that integrate transcriptomic data with multi-level biomedical information. However, their performance heavily relies on the quality of the prior knowledge networks, limiting generalizability, especially for novel compounds or targets.

Recent deep learning methods have advanced transcriptome-based prediction by learning informative representations from high-dimensional perturbation data. SSGCN (Zhong *et al.* 2022) uses spectral-based GCN to align perturbation transcriptomic data with the protein-protein interaction (PPI) network, learning feature representations at the network level. Compared to traditional similarity-based methods, SSGCN achieves significant improvements. FRoGS (Chen *et al.* 2024), on the other hand, projects gene sets into a functional embedding space using a word2vec-inspired framework. Both studies demonstrate the effectiveness of considering inter-gene dependencies for DTIs prediction using perturbation transcriptomics. However, these methods still have limitations. In conventional graph-based models such as SSGCN, genes are represented as nodes and gene-gene associations are modeled as pairwise edges. This pairwise formulation limits the explicit modeling of higher-order functional relationships, in which multiple genes jointly participate in the same biological process or regulatory program. Meanwhile, FRoGS primarily focuses on gene-set embeddings and does not fully utilize the specific differential expression values induced by perturbations, which may limit its classification performance and increase false positive predictions.

To address this challenge, we propose the Biological function-guided Hypergraph Siamese Network (BioHSNet), a deep learning framework designed for DTIs prediction. In BioHSNet, Gene Ontology (GO) (Ashburner *et al.* 2000) terms are used to define biologically meaningful hyperedges, with each hyperedge connecting all genes annotated to the corresponding GO term. Unlike pairwise graph representations, this GO-guided hypergraph preserves functional gene groups as higher-order units rather than decomposing them into isolated gene-gene edges. This design enables BioHSNet to aggregate perturbation signals across multiple functionally related genes and to capture many-to-many gene relationships defined by curated GO knowledge. Furthermore, BioHSNet integrates compound structural features by encoding them through pre-trained graph transformer (Li *et al.* 2022), which are then fused with transcriptomic embeddings. It should be noted that the derived embeddings provide complementary chemical information rather than direct dependence on task-specific biomedical knowledge graphs. This bridges the gap between structural and functional perspectives, enhancing the model’s ability to predict DTIs with high precision. Systematic evaluations on the LINCS Phase I and Phase II datasets, demonstrate that BioHSNet outperforms baseline methods in terms of both accuracy and generalization. The case study further demonstrates its practical utility for target prediction and drug screening.

## 2 Methodology

### 2.1 The overview of BioHSNet

**Figure 1:**
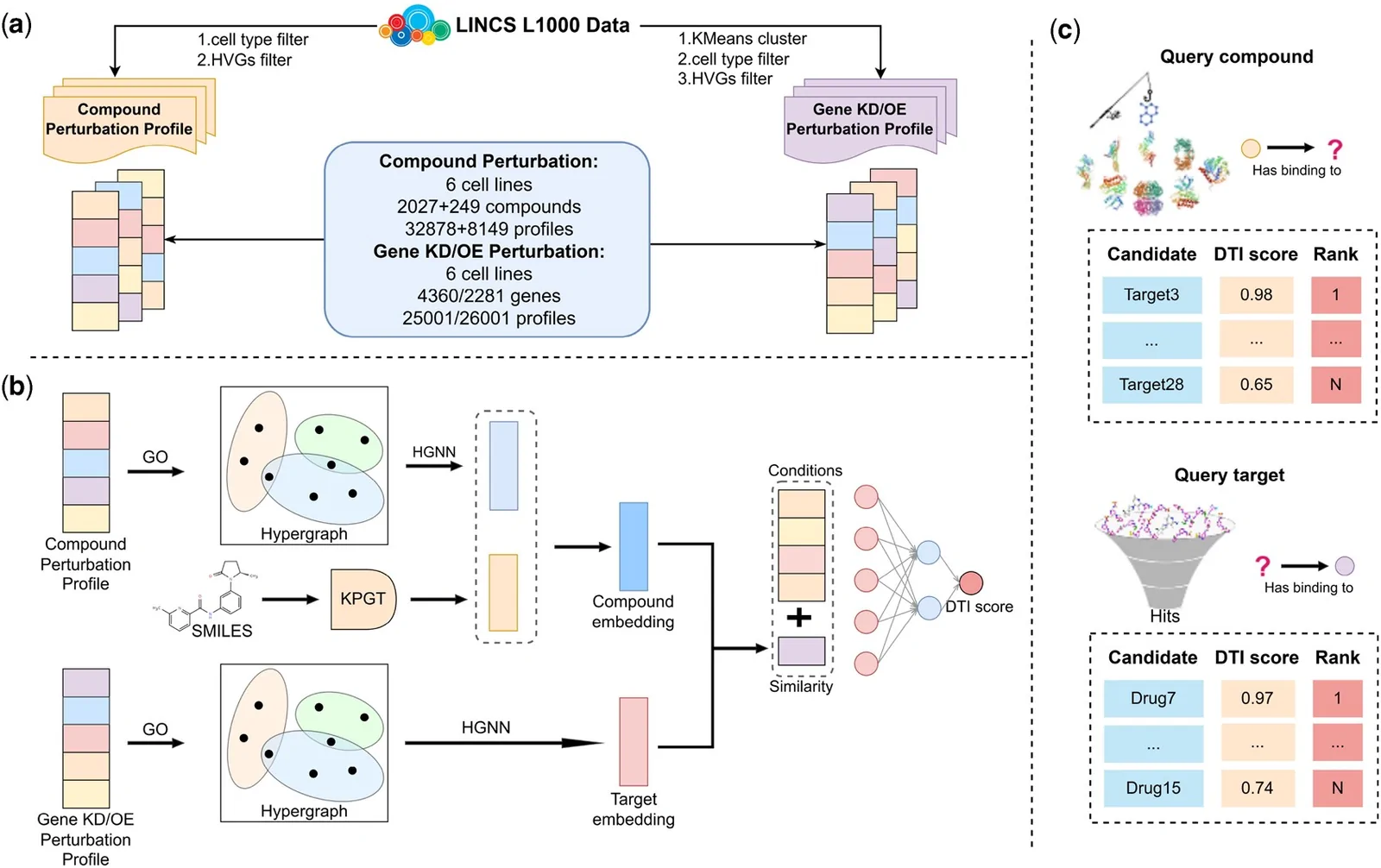
The illustration of BioHSNet. (a) Data processing flow for BioHSNet. (b) Architecture of BioHSNet. Compound embedding is obtained by incorporating both graph-based embeddings from the hypergraph and structural embeddings. Target embedding is obtained by HGNN to integrate the GO terms and gene knockdown/overexpression perturbation profile. Experimental conditions include cell type, compound dosage, compound perturbation time and gene perturbation time, and they are combined with the similarity score as input to the predictor. (c) The inference stage of BioHSNet.

The architecture of BioHSNet is illustrated in Fig. 1b. To capture inter-gene biological dependencies, BioHSNet projects transcriptomic features from gene and compound perturbations onto hypergraphs instantiated with GO terms. This yields two parallel hypergraphs, one for compound perturbations and one for gene perturbations, reflecting the functional landscape of the underlying perturbations. The gene perturbation profiles can either represent gene knockdown, corresponding to inhibitory interactions, or gene overexpression, which reflects activation effects. Then, a Hypergraph Neural Network (HGNN) (Feng *et al.* 2019) is employed to aggregate multi-gene neighborhood information and learn low-dimensional embeddings that represent the functional topology of the gene space. We can obtain hypergraph embeddings for both compound perturbation and gene perturbation. For compound perturbations, molecular structural features are incorporated to enhance biological relevance, with structural representations extracted from a pre-trained Knowledge-guided Graph Transformer (KPGT) (Li *et al.* 2022) and concatenated with hypergraph-derived features to form the comprehensive compound embedding. Finally, BioHSNet computes the cosine similarity between compound and gene embeddings to estimate interaction likelihoods. Experimental metadata such as cell type, dosage, and perturbation time are incorporated as auxiliary inputs to a fully connected predictor that outputs binary interaction probabilities.

During inference (Fig. 1c), we can query BioHSNet with a compound or target of interest such as target inference or ligand virtual screening. Following the query, BioHSNet calculates the DTI scores using the trained model and generates a recommended list based on these scores. The details of methods are as follows.

### 2.2 Hypergraph siamese network

We begin by mapping both compound perturbation data and gene perturbation data into their respective hypergraph networks based on GO. For each perturbation profile, the differential expression values for genes are used as node attributes. Let $G=\{g_{1},g_{2},\ldots ,g_{m}\}$ represent the set of genes, where *m* is the number of genes, and $D=\{d_{1},d_{2},\ldots ,d_{m}\}$ represent the differential expression values for each gene. Each GO biological process term defines a hyperedge $e_{i}$, connecting all genes involved in that process. This defines a GO-induced hypergraph $H=(G,\{e_{1},e_{2},\ldots ,e_{n}\})$, with each hyperedge $e_{i}\subseteq G$ representing a set of genes involved in the *i*-th biological process. Then two hypergraphs are constructed: one for compound perturbation $H_{c}$, and one for gene perturbation $H_{g}$, with both networks based on GO annotations. Perturbation profiles are encoded as node attributes in each hypergraph, enabling us to model the perturbation-driven gene expression responses. To extract low-dimensional embeddings that capture both gene expression responses and functional-level regulatory structure, we utilize the HGNN.

*Hypergraph Representation and Normalization:* The hypergraph is represented by an incidence matrix $H\in R^{\left|G|\times |E\right|}$, where each entry h(v,e) is 1 if gene *v* is associated with hyperedge *e* (i.e. if the gene is part of a specific GO term) and 0 otherwise. The degree of a vertex *v* is defined as the number of hyperedges it participates in, and the degree of an edge *e* corresponds to the number of genes involved in that edge. Using these definitions, we compute the normalized hypergraph adjacency matrix A, which captures gene interactions within each GO term:


(1) $$A=D_{v}^{-1/2}HWD_{e}^{-1}H^{⊤}D_{v}^{-1/2}$$

where $D_{v}$ is the node degree matrix, $D_{e}$ is the edge degree matrix, and *W* is a learnable diagonal matrix of hyperedge weights.

*Hypergraph Convolution and Embedding Learning:* The key to HGNN’s effectiveness lies in its ability to perform convolutional operations over the hypergraph structure. Given the hypergraph signal $X^{\left(l\right)}\in R^{N\times C_{l}}$ at layer *l*, the convolution operation is expressed as:


(2) $$X^{\left(l+1\right)}=\sigma \left(AX^{\left(l\right)}\Theta^{\left(l\right)}\right)$$

where $\Theta^{\left(l\right)}\in R^{C_{l}\times C_{l+1}}$ is a learnable weight matrix and σ is a non-linear activation function. The operation aggregates information from neighboring nodes (genes) within the hypergraph, capturing higher-order relationships between genes involved in similar biological processes. From this process, we can obtain gene perturbation embeddings $h_{g}$ and compound perturbation embeddings $h_{c}$.

*Incorporating Compound Structural Information:* For compound perturbations, we incorporate molecular structural features to enhance the biological relevance of the embeddings. The SMILES string for each compound is encoded using a pre-trained KPGT model to capture its structural features. These structural embeddings $e_{c}$ are then concatenated with the compound perturbation embeddings $h_{c}$ to form the comprehensive compound embedding:


(3) $$e_{c}^{\text{com}}=[e_{c}||h_{c}]$$

This fusion of structural and transcriptomic data bridges the gap between chemical structure and biological function, allowing the model to capture the complex interplay between compound properties and gene expression responses.

### 2.3 Prediction and DTI score

To predict DTIs, we compute the cosine similarity between the compound embedding $e_{c}^{\text{com}}$ and the gene perturbation embedding $h_{g}$, which serves as a measure of the interaction likelihood:


(4) $$\mathrm{Sim}(e_{c}^{\text{com}},h_{g})=\frac{e_{c}^{\text{com}}\cdot h_{g}}{\left||e_{c}^{\text{com}}||||h_{g}|\right|}$$

To account for experimental variability, we incorporate additional features such as cell type, compound dosage, and perturbation time as auxiliary features F. These features are combined with the similarity score in the final prediction model:


(5) $$S_{\text{input}}=[\text{Sim}(e_{c}^{\text{com}},h_{g})||F]$$

The prediction model consists of a fully connected layer, and the softmax function is applied to compute the DTI score:


(6) $${\text{DTI}}_{\text{score}}=\text{softmax}(\sigma (W_{1}\cdot S_{\text{input}}))$$

### 2.4 Training and optimization

The model is trained using the binary cross-entropy loss function:


(7) $$L=-y\;\text{log}(\hat{y})-(1-y)\;\text{log}(1-\hat{y})$$

where y∈{0,1} is the ground truth label, $\hat{y}$ is the prediction label. We use the Adam optimizer to minimize the loss on the training dataset. Training proceeds until convergence or early stopping based on validation performance.

### 2.5 Evaluation

To evaluate performance, we adopt the following metrics. Accuracy: overall correctness of predictions. Precision: fraction of correct positive predictions. Recall: fraction of actual positives correctly predicted. F1 Score: harmonic mean of precision and recall. AUPRC: area under the Precision-Recall curve. Top-k Accuracy: the proportion of compounds whose true targets are successfully ranked within the top-k predicted candidates.

## 3 Results

### 3.1 Overall performance

Using the LINCS Phase I dataset, we compared BioHSNet with baseline models under three settings: warm start, drug cold start, and target cold start. Experimental results (Table 1) show that BioHSNet performs strongly across all three settings, with particularly clear advantages in the drug cold start and target cold start scenarios. Under the warm start condition, BioHSNet achieves the best results in Accuracy, Precision, and F1 score. In the more challenging cold start settings, although all models experience some performance decline, BioHSNet remains more robust and generalizable. It consistently outperforms SSGCN (Zhong *et al.* 2022) and FROGS (Chen *et al.* 2024) across most metrics. All trainable baselines were evaluated under the same protocol as BioHSNet, with implementation details summarized in Table S2. Since the original FRoGS study mainly emphasized recall-oriented target recovery, we additionally assessed it using multiple classification metrics. FRoGS achieved high Recall but low Precision, F1 score, and AUPRC, suggesting severe false-positive bias. Therefore, its performance was further examined using Top-k Accuracy to assess target-ranking ability.

**Table 1:** Performance of BioHSNet and other models on classification evaluation metrics.

Metric	Warm start			Drug cold start			Target cold start		
	SSGCN	FRoGS	BioHSNet	SSGCN	FRoGS	BioHSNet	SSGCN	FRoGS	BioHSNet
Accuracy	0.9187	0.1578	**0.9294**	0.8986	0.1365	**0.9257**	0.9065	0.3021	**0.9226**
Precision	0.7607	0.0136	**0.8061**	0.8231	0.0006	**0.9613**	0.8932	0.0044	**0.9443**
Recall	**0.9847**	0.9695	0.9447	0.7571	**0.8909**	0.7324	0.7109	**0.7355**	0.7338
F1 Score	0.8583	0.0268	**0.8700**	0.7887	0.0012	**0.8314**	0.7917	0.0088	**0.8259**
AUPRC	0.9541	0.1885	**0.9549**	0.8594	0.0015	**0.9226**	0.8337	0.0045	**0.8792**

Bold values indicate the best performance for each metric under each evaluation setting.

**Figure 2:**
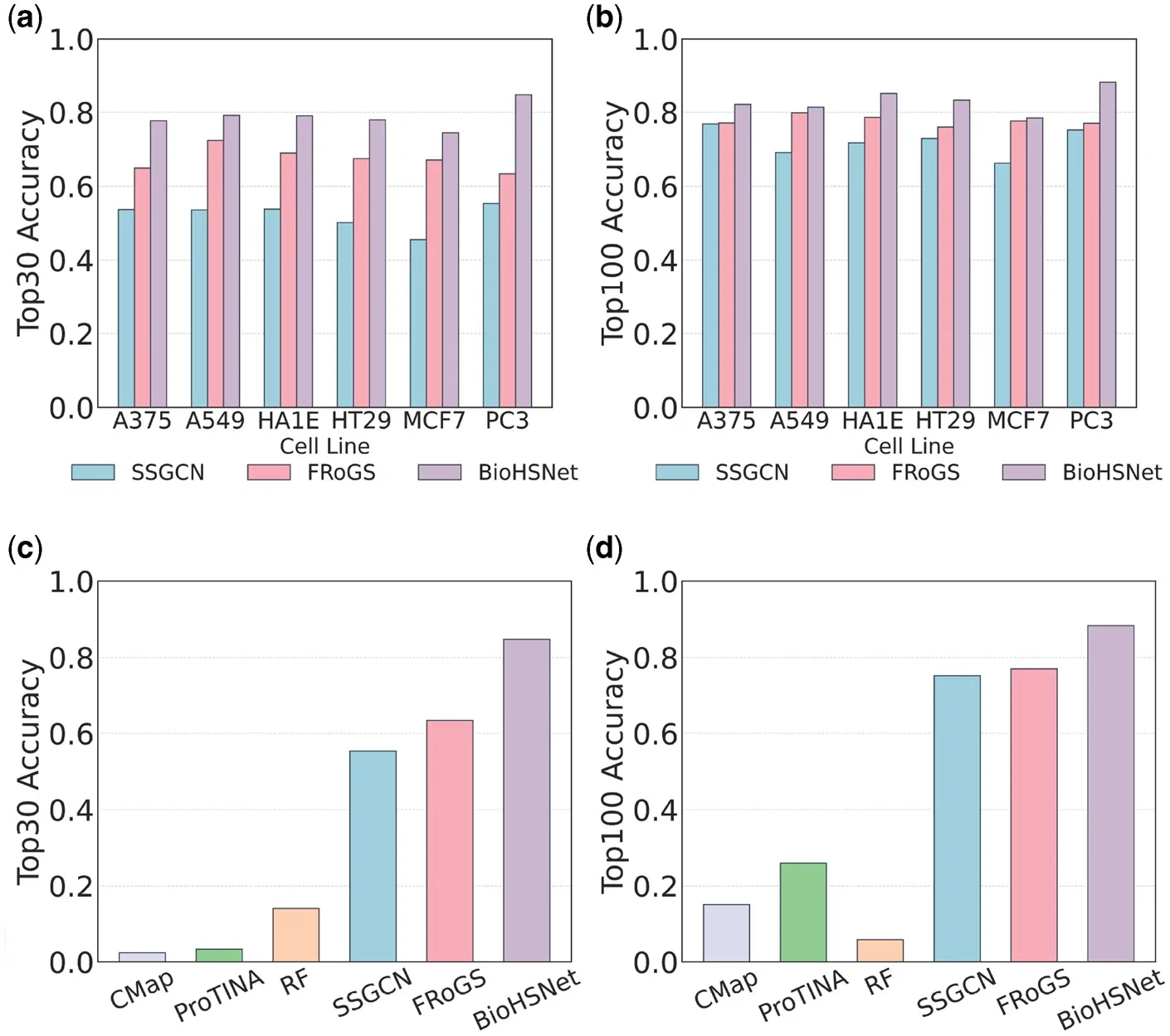
(a, b) Performance comparison of BioHSNet with two deep learning models across different cell lines. (c, d) Performance comparison of BioHSNet with baseline models on PC3 cell line.

As shown in Fig. 2a and b, BioHSNet consistently outperformed both SSGCN and FRoGS in both metrics, indicating better reliability and generalization in practical applications. For Top-30 Accuracy, BioHSNet achieved the best results across all cell lines, reaching 0.8475 in PC3, 0.7916 in A549, and 0.7904 in HA1E. Similarly, it maintained better performance in Top-100 Accuracy across all cell lines. Furthermore, as shown in Fig. 2c and d, we examined the performance of BioHSNet in comparison with CMap (Subramanian *et al.* 2017), ProTINA (Noh *et al.* 2018), and RF (Pabon *et al.* 2018) using PC3 as the example cell line. These results clearly demonstrate that BioHSNet outperforms all other models in both Top-30 and Top-100 Accuracy. We also conducted cross-cell line experiments by splitting the dataset based on cell lines (Fig. S1a and b). The results showed that BioHSNet achieved better accuracy, indicating its superior generalization ability and stronger adaptability to diverse biological contexts compared to SSGCN.

We conducted ablation experiments to assess the contributions of KPGT-derived compound structural embeddings and the GO-guided hypergraph. Two variants were designed by removing compound structural embeddings or the GO hypergraph, while keeping the other components unchanged. As shown in Fig. S1c–f, the full BioHSNet consistently outperformed both variants, especially w/o GO hypergraph. These results indicate that the GO-guided hypergraph is important for modeling functional gene relationships, and compound structural embeddings provide complementary chemical information to improve prediction performance.

### 3.2 External test evaluation

To further evaluate generalization, we performed external testing on the LINCS Phase II dataset, using SSGCN as the directly comparable graph-based baseline for the target-ranking workflow. FRoGS was not included in this analysis because it exhibited a severe imbalance between recall and precision in our Phase I experiment. As shown in Table 2, BioHSNet demonstrated stable performance on the external test set, with top-100 accuracy ranging from 0.60 to 0.67 across the six cell lines. Although there was a slight decrease in accuracy compared to the tests conducted using Phase I data, this change may be attributed to differences in the target space coverage, as well as batch effects caused by factors such as temperature, humidity, and laboratory technicians (Zhong *et al.* 2022, Arevalo *et al.* 2024). Overall, the prediction results of the BioHSNet model still showed high rationality and consistency. In contrast, the SSGCN model exhibited lower accuracy in drug target prediction, ranging from 0.39 to 0.45, indicating that BioHSNet significantly outperforms SSGCN in terms of generalization ability.

**Table 2:** Target prediction performance on the LINCS Phase II data.

Cell line	Number of compounds	BioHSNet	SSGCN	BioHSNet	SSGCN
		(Top100 acc)	(Top100 acc)	(Top30 acc)	(Top30 acc)
A375	249	**0.6346**	0.4516	0.5503	0.2321
A549	47	**0.6066**	0.4338	0.5294	0.2537
HA1E	244	**0.6415**	0.4580	0.5646	0.2500
HT29	244	**0.6729**	0.4381	0.5728	0.2271
MCF7	249	**0.6178**	0.3916	0.5482	0.2112
PC3	249	**0.6389**	0.3977	0.5518	0.2109

Bold values indicate the best performance.

Our model successfully predicted the true targets within the top-ranked predictions. For instance, the only true target of adenosine (BRD-K01577834) (Jia *et al.* 2024) was predicted at rank 11 (Fig. S2a), all 11 true targets of SB-939 (Chu *et al.* 2015) (BRD-K86797399) were predicted within the top 18 ranks (Fig. S2b). We further performed a literature search for the potential targets of these external test compounds. For example, LYN ranked first among the potential targets of saracatinib (BRD-U07805514) (Fig. S2c), and literature research revealed that the IC50 value of saracatinib against LYN is 5 nM (Green *et al.* 2009). Similarly, CDK2 ranked third among the potential targets of dinaciclib (BRD-K13662825) (Fig. S2d), with an IC50 value of 1 nM (Oner *et al.* 2025). This literature evidence further supports the strong generalizability of BioHSNet in drug target prediction.

### 3.3 Predicting potential targets for Fasudil using BioHSNet

Fasudil (BRD-K76617868) is a selective Rho-associated protein kinase (ROCK) inhibitor with established clinical use in cerebral vasospasm and related disorders (Swanson *et al.* 2017). To explore its broader pharmacological potential, we applied BioHSNet to predict candidate targets and validated the results through enrichment analysis and molecular docking.

**Figure 3:**
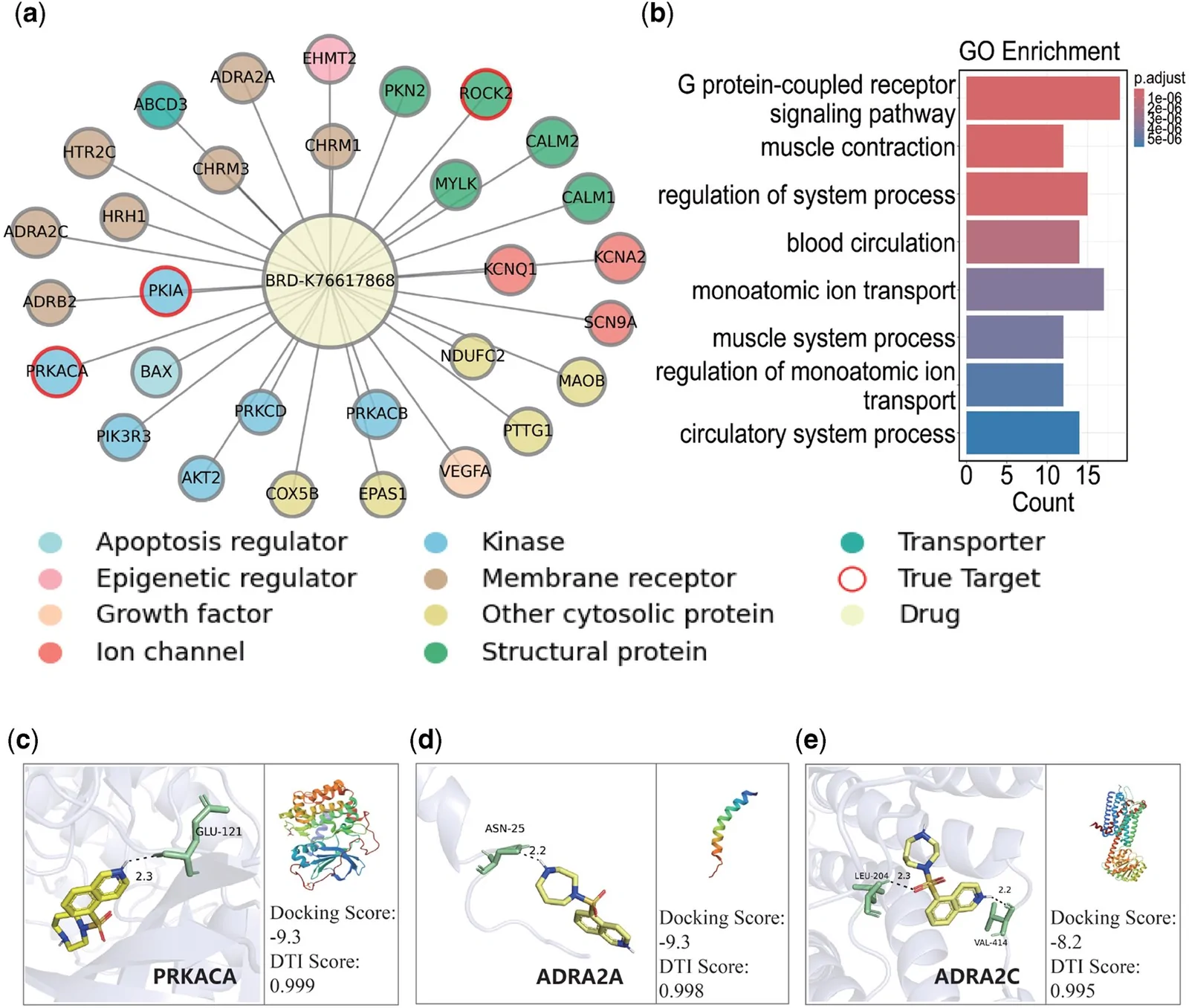
Predicting potential targets for Fasudil using BioHSNet. (a) Top 30 targets predicted by BioHSNet. (b) GO enrichment analyses for the top 30 predicted targets. (c–e) Molecular docking results.

Our results showed that BioHSNet successfully identified 3 out of 4 known targets of Fasudil within the top 7 predicted targets (Figs. 3a and S3a): PRKACA (Rank 1), PKIA (Rank 2), and ROCK2 (Rank 7). The remaining known target, ROCK1, ranked 41st, indicating that BioHSNet has a high target recognition capability. GO biological process enrichment analysis of the top 30 predicted targets (Fig. 3b) revealed significant enrichment in muscle contraction- and blood circulation-related processes, in line with Fasudil’s known vascular effects. KEGG pathway analysis (Fig. S3b) further identified the vascular smooth muscle contraction and cGMP-PKG signaling pathways, further supporting the mechanistic relevance of the predicted targets.

To further assess the functional relevance of the predicted targets, we performed a functional module-level validation analysis. As shown in Fig. S3c and Table S3, Fisher’s exact test with FDR correction confirmed significant associations between the predicted targets and Fasudil-related functional modules. These results provide additional functional evidence supporting the biological plausibility of BioHSNet predictions. Molecular docking was further performed. PRKACA, a known target of Fasudil, was included as a reference and showed a strong docking interaction with Fasudil, with a docking score of -9.3 and a predicted DTI score of 0.999 (Fig. 3c). Among the novel predicted targets, ADRA2A showed a docking score of -9.3 and a predicted DTI score of 0.998 (Fig. 3d), while ADRA2C showed a docking score of -8.2 and a predicted DTI score of 0.995 (Fig. 3e). These docking results provide additional structural support for the potential interactions between Fasudil and the predicted targets.

### 3.4 Predicting potential inhibitors for TGFBR1 using BioHSNet

Transforming growth factor-beta receptor 1 (TGFBR1) is a key component of the TGF-β signaling pathway, regulating cell proliferation, differentiation, apoptosis, and immune responses. Aberrant activation of TGFBR1 is closely linked to cancer progression, fibrosis, and immune escape (Loeys *et al.* 2005). Thus, identifying potential TGFBR1 inhibitors is of valuable therapeutic interest (Li *et al.* 2025).

**Figure 4:**
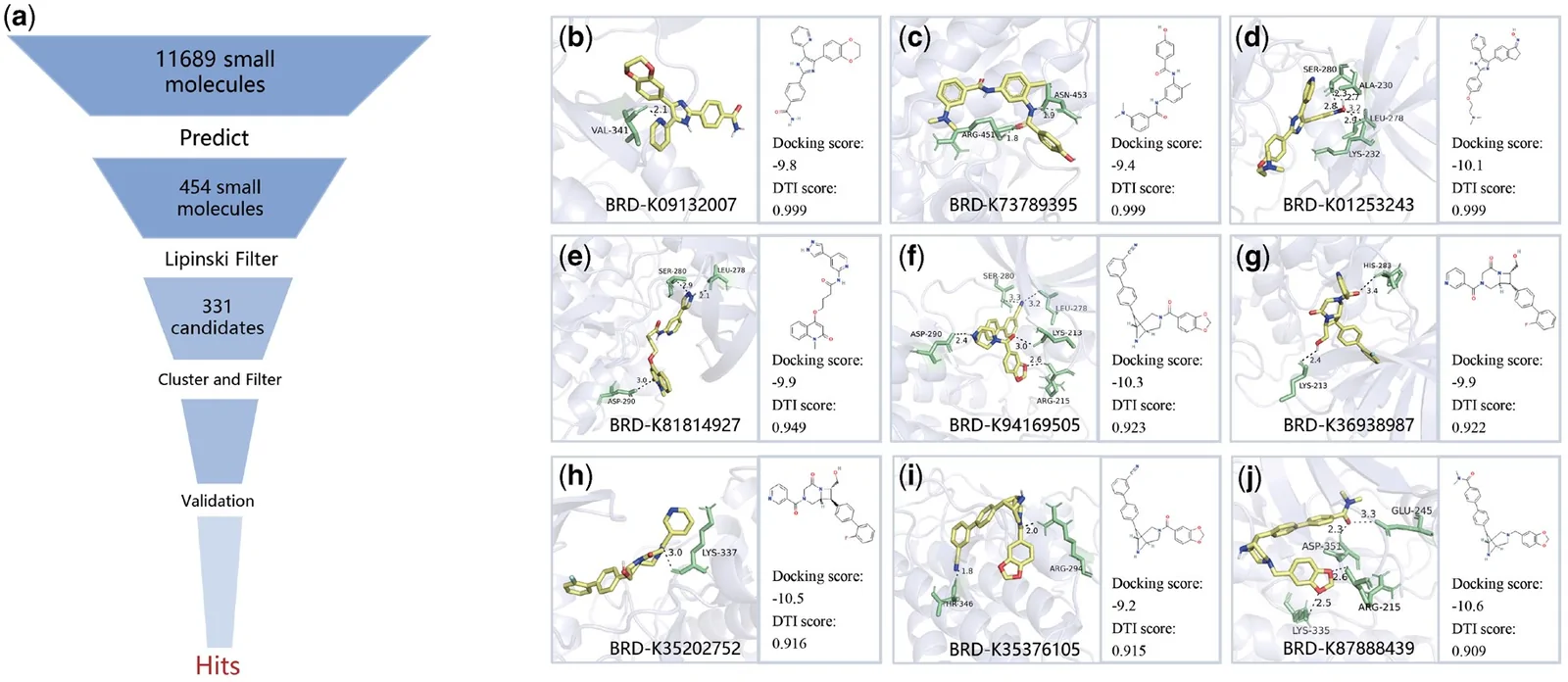
Predicting potential inhibitors for TGFBR1 using BioHSNet. (a) Screening process for potential inhibitors of TGFBR1. (b–j) Binding conformations of nine candidate compounds with strong binding affinity to the TGFBR1 active site. Hydrogen bonds and interaction distances (in Å) between ligands and critical residues are indicated with dashed lines.

We used BioHSNet to predict potential TGFBR1 inhibitors, with the screening workflow shown in Fig. 4a. Using 22 705 perturbation profiles from 11 689 compounds in the LINCS PC3 dataset, compounds were ranked by DTI score. Applying a cutoff of 0.5 yielded 454 candidate compounds. Among these, four known TGFBR1 inhibitors ranked within the top 10, specifically ranked 1st, 2nd, 3rd, and 6th (Supplementary Data 1), supporting the effectiveness of BioHSNet.

To further prioritize drug-like candidates, we applied Lipinski’s rules, reducing the set to 331 compounds. Fingerprint-based clustering was then used to improve diversity and select 53 representative compounds for molecular docking against TGFBR1. Docking identified 9 candidates with strong binding affinity (docking score < −9.1) and high predicted DTI scores (> 0.9), suggesting their potential as TGFBR1 inhibitors. These compounds formed key interactions with residues in the TGFBR1 active site. For example, BRD-K09132007 (Fig. 4b) formed a hydrogen bond with VAL-341 and achieved a docking score of −9.8, while BRD-K01253243 (Fig. 4d) formed multiple hydrogen bonds with SER-280, ALA-230, LYS-232, and LEU-278, with a docking score of −10.1. BRD-K94169505 (Fig. 4f) and BRD-K87888439 (Fig. 4j) showed even stronger binding, with docking scores of −10.3 and −10.6, respectively. The predicted DTI scores of these top compounds ranged from 0.909 to 0.999, indicating high confidence in their interactions with TGFBR1.

BioHSNet not only successfully ranked known inhibitors at the top but also uncovered structurally diverse, high-affinity compounds with drug-like properties. These candidate inhibitors could serve as promising leads for anticancer drug development targeting the TGF-β pathway.

### 3.5 BioHSNet enables prediction of both inhibitory and activatory DTIs

**Figure 5:**
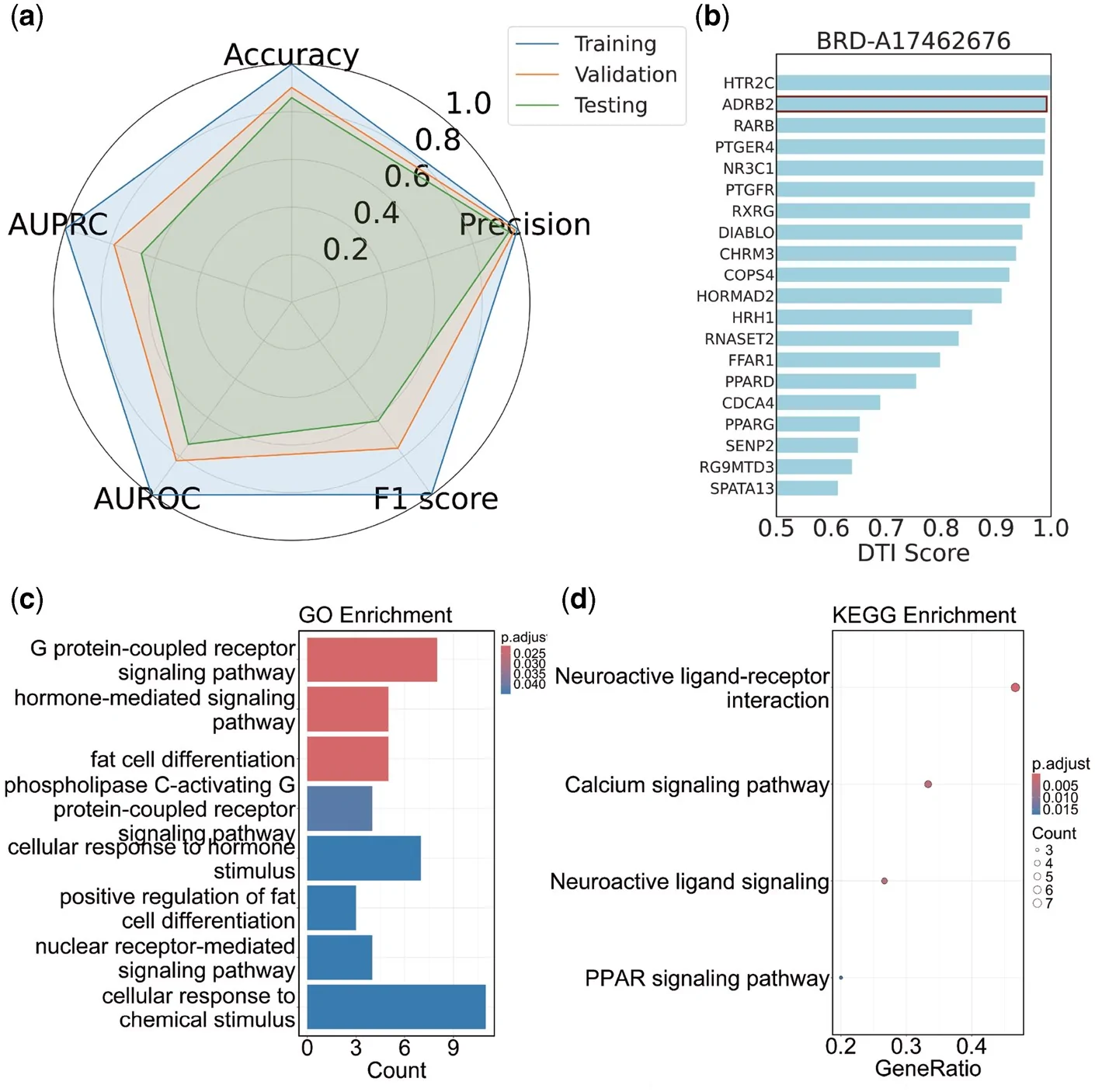
BioHSNet Predicts Drug-Target Activatory Interactions. (a) BioHSNet Performance in Activation Prediction. (b) The predicted targets of Bambuterol (BRD-A17462676) using BioHSNet. The corresponding true targets are highlighted with red borders. (c, d) GO and KEGG enrichment analyses for the predicted targets of the drug Bambuterol (BRD-A17462676).

Although the above experiments primarily focused on predicting drug-target inhibition using gene knockdown perturbation profiles, the BioHSNet framework is inherently capable of predicting both inhibitory and activatory interactions (Fig. 1b). By replacing the knockdown perturbation profiles with gene overexpression (OE) perturbation profiles, BioHSNet can predict activation effects between drugs and targets (Fig. S4a). Here, we present an example analysis of drug-target activation. As shown in the radar chart (Fig. 5a), BioHSNet achieved generally good performance across evaluation metrics when using OE perturbation profiles.

Furthermore, we used BioHSNet to predict the potential targets of Bambuterol (Groo *et al.* 2025), a long-acting $\beta_{2}$-adrenoceptor (ADRB2) agonist used in the treatment of asthma. According to the prediction results (Figs. S4b and 5b), the model successfully identified the true target, ADRB2, ranking it second among all predicted targets, demonstrating the model’s effectiveness. GO enrichment analysis of the predicted targets (Fig. 5c) showed significant enrichment in G protein-coupled receptor signaling, which is consistent with Bambuterol’s known mechanism of action. Enrichment in hormone-mediated signaling and cellular response to hormone stimulus also supports its role in smooth muscle relaxation and bronchodilation, both of which are key processes in asthma treatment. The KEGG pathway enrichment analysis (Fig. 5d) identifies significant enrichment in neuroactive ligand-receptor interactions and neuroactive ligand signaling, suggesting that Bambuterol may also influence neural pathways, though its primary clinical use is for treating respiratory conditions.

In addition, we used BioHSNet to predict potential activators of ESR1 (Dustin *et al.* 2019), which encodes estrogen receptor alpha. We evaluated DTI scores between ESR1 and 11 689 compounds, and all eight known activators were identified within the top 80 predictions (Supplementary Data 2). This result further suggests that BioHSNet remains effective in activation-related prediction tasks.

## 4 Discussion

In this study, we have proposed BioHSNet, a flexible hypergraph siamese framework that integrates perturbation transcriptomics with compound structure to predict DTIs. BioHSNet captures higher-order dependencies in biological systems and integrates structural and functional perspectives, enabling robust predictions from both chemical properties and transcriptomic responses. Our results demonstrate that BioHSNet outperforms baseline methods across multiple evaluation metrics, with particularly superior performance observed in cold start scenarios. The superior performance of BioHSNet in external validation experiments highlights its generalizability. Case studies highlight BioHSNet’s strong predictive ability in both target discovery and compound prioritization, offering a reliable computational strategy to accelerate drug repurposing and lead optimization. In addition, BioHSNet can predict both inhibitory and activatory DTIs using gene knockdown or overexpression perturbation profiles, respectively, thereby broadening its applicability across therapeutic contexts.

However, several aspects can be further explored in future work. BioHSNet does not directly rely on task-specific biomedical knowledge graphs for DTIs inference, but uses the KPGT encoder to extract compound structural embeddings. These embeddings provide complementary chemical information rather than direct compound-target relation information, enriching the perturbation-based representation from a structural perspective. However, they may still be influenced by the chemical space covered during pretraining. Therefore, future work will further optimize molecular encoders to better handle compounds with rare scaffolds or unusual structures. In addition, incorporating temporal or single-cell profiles may further improve prediction specificity and biological interpretability.

## Supplementary Material

btag475_Supplementary_Data

## Data Availability

The level 5 perturbation profiles from the L1000 CMap (Subramanian *et al.* 2017) dataset were downloaded from the Gene Expression Omnibus (GEO) database (accession numbers: GSE92742 and GSE70138) via https://www.ncbi.nlm.nih.gov/geo. The data preprocessing process is illustrated in Fig. 1a, while detailed descriptions of the procedures are provided in the Supplementary Materials. The human Gene Ontology data was downloaded from https://current.geneontology.org. The compound structural data used in this study were obtained from https://www.ebi.ac.uk/chembl. The source code is available at https://github.com/Zxinyizhang/BioHSNet and has been archived on Zenodo with the DOI: https://doi.org/10.5281/zenodo.20457122.
