# Supplementary material for: Drug target prediction from perturbation transcriptomics via a biological function-guided hypergraph siamese network
Source: Bioinformatics. 2026 Aug 6;42(8):btag475. doi: 10.1093/bioinformatics/btag475 (PMC13452056; doi:10.1093/bioinformatics/btag475)
Supplement: Supplementary file 1 [file btag475_supplementary_data.zip › Supplementary_information.pdf]

Supplememtary Information

Drug Target Prediction from Perturbation Transcriptomics via a Biological Function-Guided Hypergraph Siamese Network

Xinyi Zhang[1,†], Xinliang Sun[1,†], JiuXu Yang[1] and Min Li[1,*]

[1] School of Computer Science and Engineering, Central South University, Changsha 410083, China

[†] These two authors contribute equally to the work.

*** Correspondence:**

Min Li

Email: limin@mail.csu.edu.cn

Keywords: Drug target prediction, Perturbation transcriptomics, Hypergraph siamese network

Supplemental Method Details

Data collection and preprocessing

The level 5 perturbation profiles from the L1000 CMap dataset were downloaded from the Gene Expression Omnibus (GEO) database (accession numbers: GSE92742 and GSE70138) via https://www.ncbi.nlm.nih.gov/geo.

We utilized the cmapPy library to filter perturbation transcriptomes for "trt_cp" (compound treatment), "trt_sh" (shRNA knockdown) and "trt_oe" (cDNA overexpression) across six cell lines (A375, A549, HA1E, HT29, MCF7 and PC3). To obtain robust shRNA knockdown signatures, we performed k-means clustering (k = 1) on the "trt_sh" signatures, separated by cell lines and knockdown time points. We then retained the core signature, i.e., the centroid of each cluster, to represent the corresponding cluster. A similar approach was applied to obtain cDNA overexpression signatures. The database contains 978 landmark genes and a total of 12,328 extended genes. To enhance the experimental efficiency, we selected 5,355 genes with high variance for further analysis. For compound perturbation, we obtained 32,878 perturbation profiles corresponding to 2,027 compounds in Phase I data, and 8,149 profiles corresponding to 249 compounds in Phase II data. For gene perturbation, we obtained 25,001 perturbation profiles corresponding to 4,360 knockdown genes and 26,001 perturbation profiles corresponding to 2,281 overexpressed genes.

Gene Ontology (GO) Data: The human Gene Ontology data was downloaded from https://current.geneontology.org. We filtered Gene Ontology (GO) terms associated with high-variance genes, resulting in 8,365 distinct GO terms.

SMILES: The compound structural data used in this study were obtained from https://www.ebi.ac.uk/chembl, a widely used open-access resource containing experimentally validated bioactive molecules.

The dataset was partitioned into training, validation, and test sets according to a predefined split ratio under three evaluation settings: warm start, drug cold start, and target cold start. In the warm-start setting, compound-target pairs were divided according to the split ratio, while compounds and targets could appear in different subsets. In the drug cold-start setting, compounds were partitioned into different subsets, and compounds in the validation and test sets were excluded from the training set. In the target cold-start setting, targets were partitioned into different subsets, and targets in the validation and test sets were excluded from the training set.

Dataset partitioning and sampling: Positive samples were defined based on known compound-target associations. Negative samples were generated by pairing compounds with targets without annotated interactions after

excluding known positive pairs. During training, negative samples were sampled at an approximately 3:1 ratio relative to positive samples. Detailed statistics for each split, including the numbers of compounds, targets, positive samples, negative samples, cell lines, and perturbation profiles, are provided in Table S1.

Supplemental Table S1. Dataset statistics under different evaluation settings.

Setting	Split	Compounds	Targets	Positive samples	Negative samples	Cell lines	Perturbation profiles
Warm start	Train	1823	698	4779	14337	6	114696
	Validation	399	261	1802	5406	6	43248
	Test	423	256	1789	5367	6	42936
Drug cold start	Train	1621	704	3954	12024	6	96192
	Validation	203	252	553	1659	6	13272
	Test	203	205	503	1509	6	12072
Target cold start	Train	1791	604	3951	11853	6	94824
	Validation	489	75	573	1719	6	13752
	Test	418	76	486	1458	6	11664

Baseline implementation

All trainable baseline methods were evaluated using the same training, validation, and test splits as BioHSNet whenever applicable. The same positive and negative DTI labels were used for model training and evaluation. Hyperparameters were selected according to validation performance, and the test set was used only for final evaluation.

SSGCN was implemented following its original graph-convolutional framework, using perturbation transcriptomic profiles and the PPI network as input. Since the original FRoGS study mainly emphasized recall-oriented target recovery, we evaluated FRoGS using multiple complementary metrics, including Accuracy, Precision, Recall, F1 score, and AUPRC. In addition, Top-30 and Top-100 Accuracy were used to evaluate target-ranking performance.

CMap, ProTINA, and RF were evaluated in the PC3 target-ranking experiment using the same candidate target ranking protocol. The implementation source, input data, and evaluation metrics for each baseline

are summarized in Table S2.

Supplemental Table S2. Baseline implementation and evaluation details.

Baseline	Implementation source	Training/tuning	Evaluation metrics
BioHSNet (ours)	https://github.com/Zxinyizhang/BioHSNet	Hyperparameters selected by validation performance	Classification metrics, Top-k accuracy
SSGCN	https://github.com/boyuezhong/SSGCN	Original optimal hyperparameters	Classification metrics, Top-k accuracy
FRoGS	https://github.com/chenhcs/FRoGS	Original optimal hyperparameters	Classification metrics, Top-k accuracy
CMap	https://clue.io/l1000-query	-	Top-k accuracy
ProTINA	https://github.com/CABSEL/ProTINA	Original optimal hyperparameters	Top-k accuracy
RF	http://sb.cs.cmu.edu/Target2/	Original optimal hyperparameters	Top-k accuracy

Functional module-level validation of predicted Fasudil targets

To further evaluate the biological plausibility of BioHSNet-predicted Fasudil targets, we performed a functional module-level validation analysis using curated MSigDB gene-set annotations (Liberzon et al., 2015). Four Fasudil-related modules were predefined based on known pharmacological mechanisms and pathway annotations: Rho/ROCK signaling and cytoskeleton regulation, vascular smooth muscle contraction (Loirand et al., 2006), cAMP/PKA signaling, and adrenergic receptor-mediated vascular regulation.

For each module, the corresponding MSigDB gene sets were collected and intersected with the BioHSNet candidate target space. After removing duplicated genes and retaining targets within the candidate target space, 28 top-ranked Fasudil targets were included in this analysis. Overall, these targets covered all four predefined Fasudil-related modules, and 18 of 28 targets were associated with at least one module. For clarity, Figure S3c shows a Sankey plot of 10 representative module-associated predicted targets. The proportion of module-associated targets was substantially higher than that observed in 10,000 randomly sampled target sets of the same size from the candidate target space (18/28 vs. random mean = 6.65, empirical $p < 1 \times 10^{-4}$).

We further performed Fisher's exact test using all candidate targets as the background, followed by FDR correction. As summarized in Supplemental Table S3, all four predefined modules were significantly overrepresented among the top-ranked Fasudil targets. These results provide additional functional evidence supporting the biological plausibility of the predicted Fasudil targets.

Supplemental Table S3. Functional module-level validation of predicted Fasudil targets.

Fasudil-related functional module	No. of mapped top targets	FDR
Rho/ROCK signaling and cytoskeleton regulation	9	9.44×10^{-4}
Vascular smooth muscle contraction	11	1.09×10^{-10}
cAMP/PKA signaling	16	1.96×10^{-6}
Adrenergic receptor-mediated vascular regulation	6	2.27×10^{-8}

Molecular docking procedure

In Section 3.3 and 3.4, the protein structures of the predicted targets were obtained from the Protein Data Bank (https://www.rcsb.org/), and the compound structure information was obtained from PubChem (https://pubchem.ncbi.nlm.nih.gov). Water molecules and heteroatoms were removed from the protein structures, followed by the addition of polar hydrogens and charge assignment. Molecular docking was performed using AutoDock 4.2.6 (Morris et al., 2009), and the docking poses with the lowest binding energy were selected for further analysis. The final docking results were visualized using PyMOL(DeLano, 2002).

Supplemental Figures

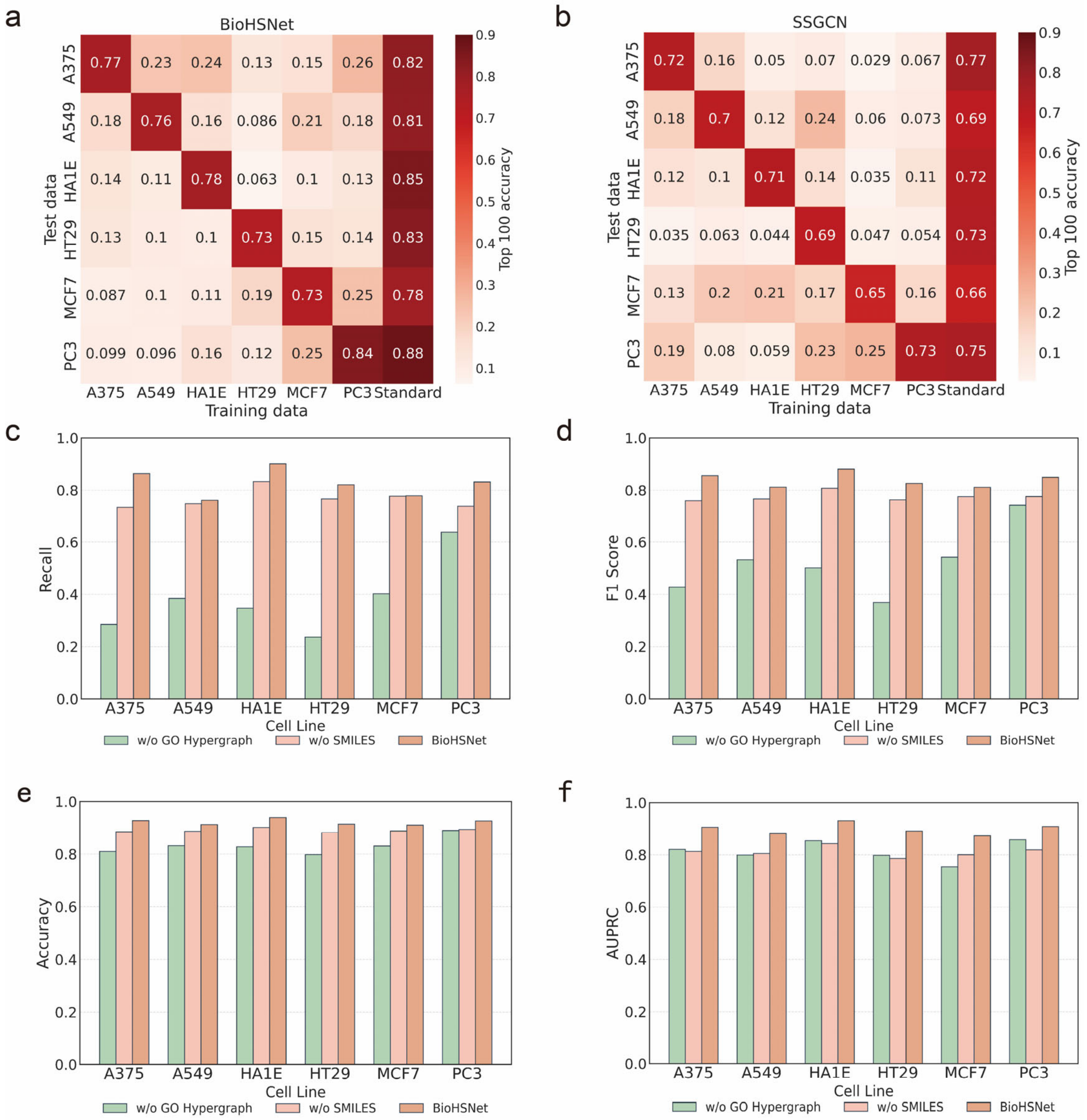

Supplemental Figure S1. (a-b) Heatmap showing cross-cell-line Top-100 Accuracy of SSGCN and BioHSNet, where each cell line represents the prediction performance when the model is trained on one cell line and tested on another; (c-f) Ablation analysis of key BioHSNet components across six cell lines.

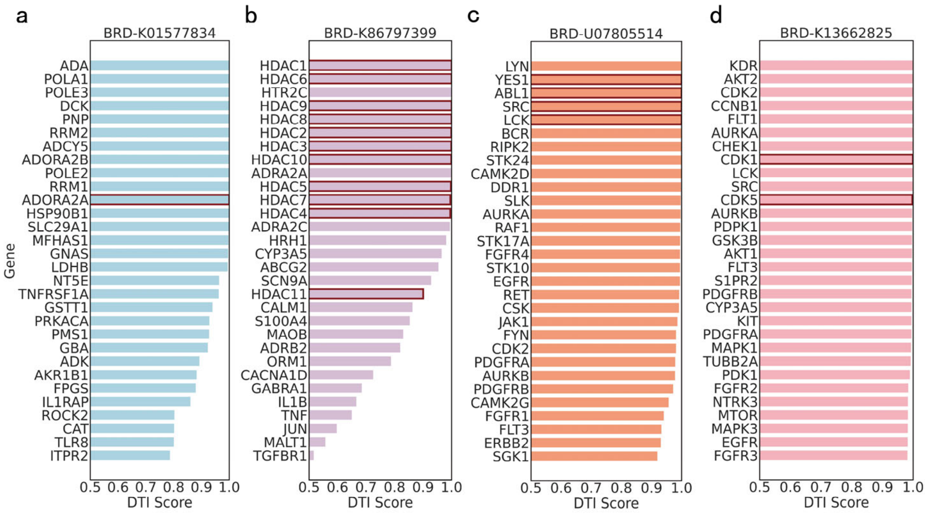

Supplemental Figure S2. Prediction of Potential Targets for External Test Compounds by BioHSNet. In each plot, the red boxes represent the known true targets for the respective compounds, showcasing BioHSNet's ability to successfully predict these targets within the top ranks.

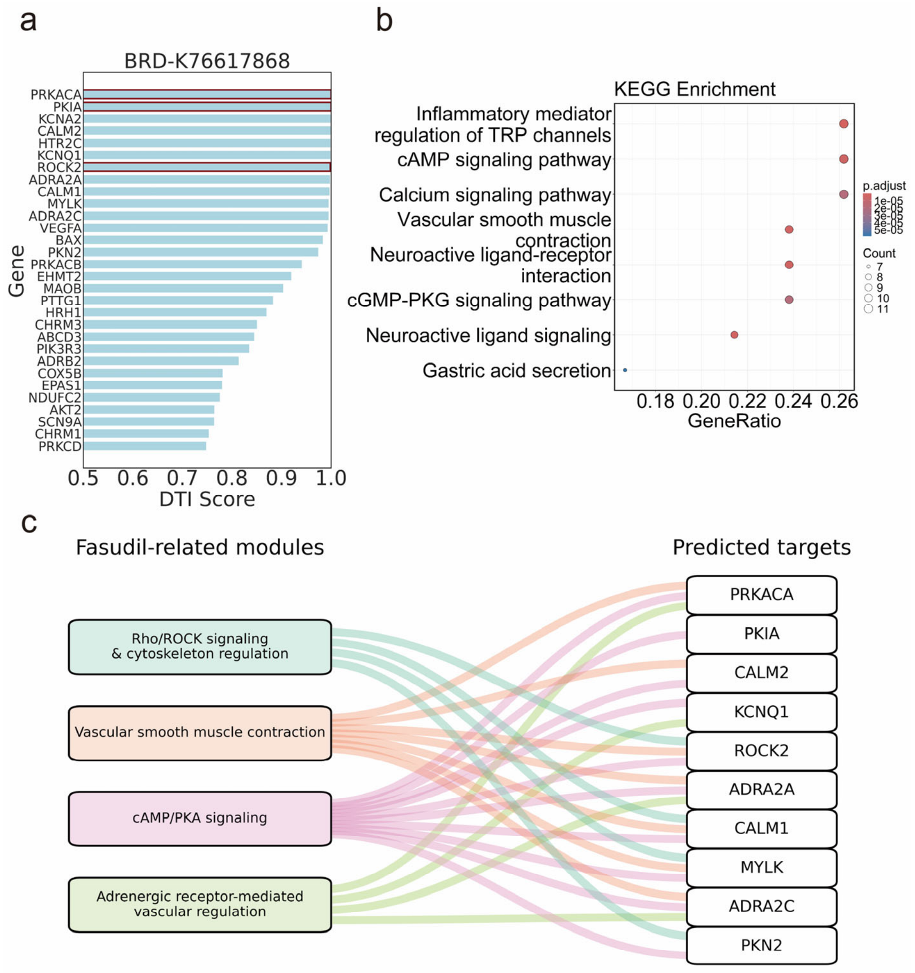

Supplemental Figure S3. (a) Predict the top 30 targets of the drug Fasudil (BRD-K76617868) using BioHSNet. The corresponding true targets are highlighted with red borders. (b) KEGG enrichment analyses for the predicted targets of the drug Bambuterol Fasudil (BRD-K76617868). (c) Functional module-level validation of predicted Fasudil targets.

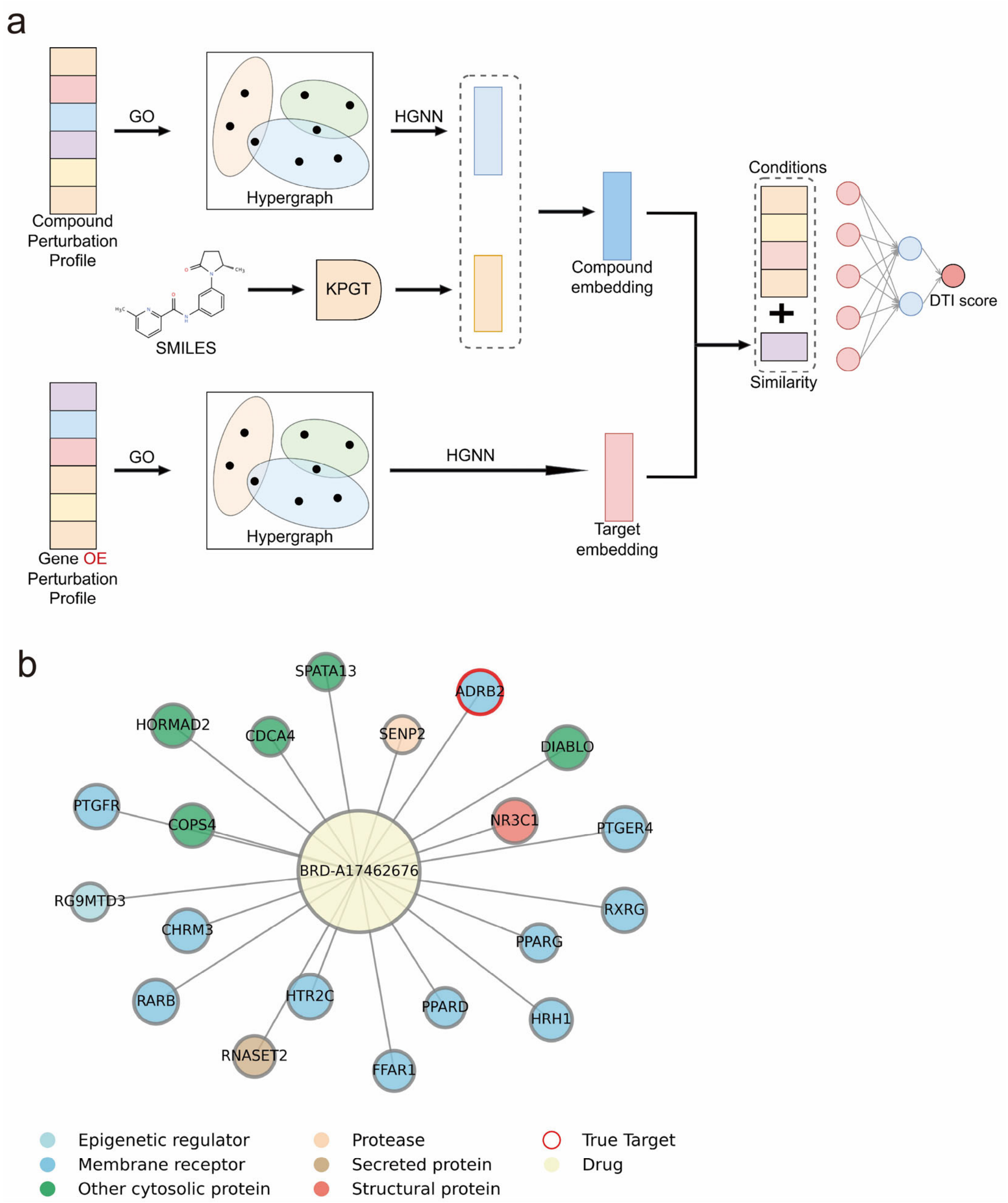

Supplemental Figure S4. (a) The workflow of BioHSNet for utilizing gene overexpression perturbation profiles to predict activatory DTIs. (b) The predicted targets of Bambuterol (BRD-A17462676) using BioHSNet . The nodes in the middle represent the compound, while the nodes in the outer circles represent the predicted targets. Predicted targets with higher scores are represented by larger circles.

Reference

DeLano, W. L. (2002). Pymol: An open-source molecular graphics tool. *CCP4 Newsl. protein crystallogr*, *40*(1), 82-92.

Liberzon, A., Birger, C., Thorvaldsdóttir, H., Ghandi, M., Mesirov, J. P., & Tamayo, P. (2015). The molecular signatures database hallmark gene set collection. *Cell systems*, *1*(6), 417-425.

Loirand, G., Guérin, P., & Pacaud, P. (2006). Rho kinases in cardiovascular physiology and pathophysiology. *Circulation research*, *98*(3), 322-334.

Morris, G. M., Huey, R., Lindstrom, W., Sanner, M. F., Belew, R. K., Goodsell, D. S., & Olson, A. J. (2009). AutoDock4 and AutoDockTools4: Automated docking with selective receptor flexibility. *Journal of computational chemistry*, *30*(16), 2785-2791.
